# Aerobic Exercise Restores Hippocampal Neurogenesis and Cognitive Function by Decreasing Microglia Inflammasome Formation Through Irisin/NLRP3 Pathway

**DOI:** 10.1111/acel.70061

**Published:** 2025-04-07

**Authors:** Renqing Zhao, Xin Tian, Haocheng Xu, Yuanxin Wang, Junjie Lin, Bin Wang

**Affiliations:** ^1^ College of Physical Education, Yangzhou University Yangzhou China

**Keywords:** aerobic exercise, cognition, FNDC5/irisin, hippocampal neurogenesis, NLRP3 inflammasome, Parkinson disease

## Abstract

Persistent microglial inflammation is a detrimental contributor to the progression of Parkinson disease (PD) pathology and related issues such as impaired adult hippocampal neurogenesis (AHN) and cognition. We conducted a 10‐week exercise program with MPTP‐treated mice to determine whether neuroinflammation can be addressed by aerobic exercise and elucidate its underlying regulatory mechanisms. Ten weeks of exercise significantly reduced PD‐related pathology and enhanced AHN and memory. These changes were linked to a reduction in neuronal apoptosis, microglial inflammation, and NLRP3 inflammasome activation. In cultured microglia, fibril α‐synuclein reduced FNDC5/irisin protein levels and induced NLRP3 inflammasome formation and IL‐1β production, which could be diminished by recombinant irisin treatment. Interestingly, “runner serum” isolated from exercising rodents enhanced FNDC5/irisin expression and reduced NLRP3 inflammasome components and IL‐1β secretion in α‐synuclein‐treated microglia. These effects could be diminished by blocking irisin signaling with cyclo RGDyk or NLRP3 agonist, nigericin sodium salt. Exercise‐induced neuroprotective effects were weakened by treatment of MPTP‐treated mice with cyclo RGDyk. In contrast, systematic administration of irisin partially replicated the beneficial effects of exercise on PD pathology, AHN, and memory function. As a nonpharmacological strategy, aerobic exercise effectively addresses PD pathology and preserves adult neurogenesis and cognition by mitigating microglial inflammation via mediating irisin/NLRP3 inflammasome pathways.

## Graphical abstract

Exercise improved PD pathology and memory by reducing microglial inflammation, apoptosis, and the formation of the NLRP3 inflammasome via irisin signaling. Irisin administration partially replicated the beneficial effects of exercise on PD pathology.

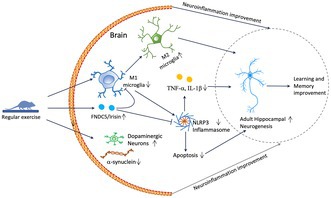

## 1 Introduction

Parkinson disease (PD) is a prevalent neurodegenerative condition characterized by the widespread presence of Lewy bodies and the loss of dopaminergic (DAergic) neurons in the central nervous system (CNS) (Zaman et al. 2021). It impacts approximately 2‐3% of people over 65 years old and presents with motor dysfunction such as bradykinesia, tremors, and rigidity (Aarsland et al. 2021). However, growing evidence has indicated that individuals with PD frequently experience mild cognitive impairment (MCI) (Aarsland et al. 2017). The incidence of MCI in PD patients is approximately seven times higher than in the general older population (Aarsland et al. 2001), with approximately 60% of these patients potentially developing Alzheimer disease (AD) within 5 years (Domellof et al. 2015). However, effective approaches for treating MCI in PD patients are currently lacking.

Recent studies have highlighted the significant role of neuroinflammation in the progression of neurodegeneration in PD (Han and Le 2023). Elevated levels of inflammatory factors, including interleukin (IL)‐1β, IFN‐γ, and IL‐6, have been observed in various regions of the brain, such as the nigrostriatum, hippocampus, cortex, and cerebrospinal fluid of PD patients (Han and Le 2023; Kouli et al. 2020). Interestingly, anti‐inflammatory or immunosuppressive interventions show the potential to mitigate damage to DAergic neurons (Kouli et al. 2020). Moreover, fibril α‐synuclein can stimulate the expression of the NOD‐like receptor family pyrin domain containing 3 (NLRP3) through Toll‐like receptor (TLR) pathways in microglial cells (Singh et al. 2023), leading to an increase in pro‐IL‐1β production. Pro‐IL‐1β can be cleaved by caspase‐1 to release mature IL‐1β (Han and Le 2023). Excessive α‐synuclein accumulation can cause specifically hippocampal neurogenic niche inflammation, where newborn neurons are continuously generated, ultimately impairing adult hippocampal neurogenesis (AHN) and cognitive function (Kouli et al. 2020). Those data suggest that PD pathology facilitates microglial NLRP3 inflammasome formation and induces persistent hippocampal inflammation, contributing to AHN and cognitive impairment. This provides a therapeutic approach to addressing MCI in PD.

A growing body of evidence has indicated that aerobic exercise has a profound impact on neuroinflammation (Aaron and Brendon 2021; de Miguel et al. 2021; Mee‐Inta et al. 2019). For example, it has been shown to promote the transformation of microglia from the M1 to M2 phenotype, causing a reduction in the release of proinflammatory factors and an improvement in hippocampal neuroinflammation (Wang et al. 2023a). The beneficial effects of exercise on microglia activation and inflammatory production lead to the improvement of AHN and cognitive function (de Almeida et al. 2022). Interestingly, injecting exercise mouse plasma into nonexercise mice can mimic the effects of exercise by inhibiting neuroinflammation and promoting AHN and memory function (de Miguel et al. 2021; Horowitz et al. 2020). However, the molecular mechanism underlying how exercise ameliorates neuroinflammation and enhances AHN and cognitive function remains unclear.

Recent evidence suggests that the capacity of exercise to promote the biological and physiological function of organs is associated with its secretion of numerous myokines, such as irisin, which is distributed through the bloodstream and exerts its functions in various organs, including the brain (Lin et al. 2024; Sun et al. 2018; Wang et al. 2024; Zhao 2022; Zhao 2024a; Zhao 2024b). Irisin can enhance neuronal plasticity and function by addressing neuroinflammation in various neurodegenerative diseases (Zhao 2022; Zhao 2024a; Zhao 2024b). Lourenco et al. (2019) investigated irisin's potential to mitigate synapse failure and memory dysfunction in AD. They found that knocking down *Fndc5 (fibronectin type III domain‐containing protein 5)* using lentiviruses with two types of shRNA constructs impaired hippocampal long‐term potentiation and memory during the novel object recognition task. Conversely, increasing *Fndc5* expression in the hippocampus restored synaptic plasticity and memory in AD mice. Notably, irisin expression is required for the neuroprotective effects of physical exercise on synaptic plasticity and memory in AD mice (Lourenco et al. 2019). Similarly, Wang et al. (2018) discovered that irisin treatment protected neurons from the toxicity of Aβ peptides and reduced the production of IL‐6 and IL‐1β in astrocyte cultures. It also decreased the expression of cyclooxygenase 2, phosphorylated protein kinase B (AKT), and nuclear factor kappa B (NF‐κB) in microglia (Wang et al. 2018). Compelling evidence suggests that irisin plays a crucial role in mediating the beneficial effects of exercise on brain plasticity and function in various neurological diseases (Zhao 2022; Zhao 2024a; Zhao 2024b). However, the specific interactions between irisin and microglial inflammation in relation to the protective effects of exercise on PD pathology remain unclear. To address these gaps, this study aimed to investigate the effects of exercise on MPTP‐related neuropathology, neuroinflammation, AHN, and memory function. Additionally, the potential role of irisin in regulating the neuroprotective effects of exercise was also examined.

## 2 Results

### 2.1 Aerobic Exercise Alleviated α‐Synucleinopathy and Rescued Hippocampal Neurogenesis and Memory

**FIGURE 1:**
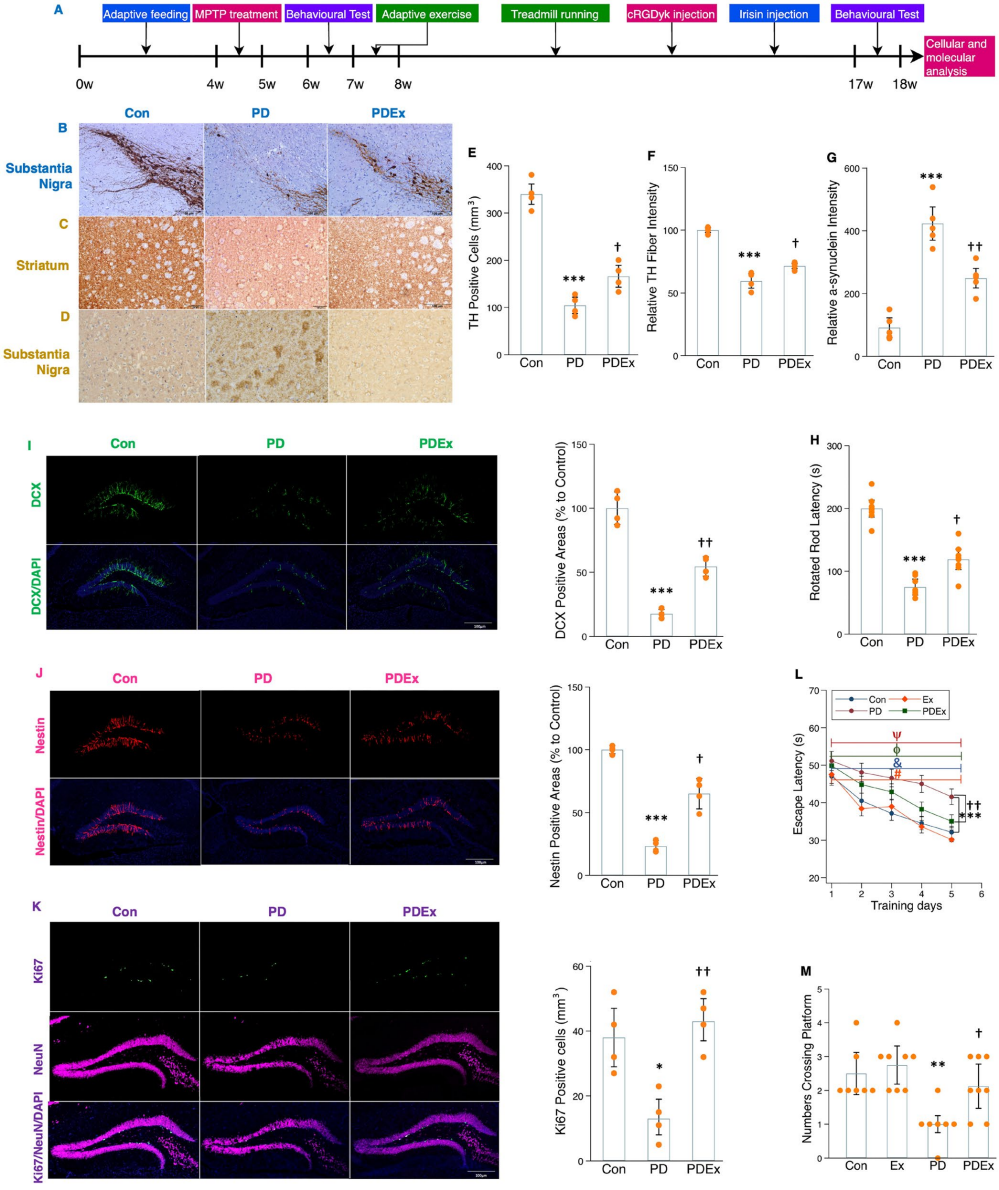
Effects of exercise on DAergic neurons, α‐synuclein, motor function, adult hippocampal neurogenesis, and memory function. (A) Timeline of experimental protocol. (B, C, and D) Immunohistochemistry of TH in substantia nigra (B), striatum (C), and α‐synuclein (D) in striatum tissue sections of mice in different groups. Scale bars, 100 μm. (E, F, and G) The histograms show the number of TH‐positive neurons (E) in the substantia nigra, the TH fiber intensity relative to the control (F) in the striatum, and the relative α‐synuclein intensity (G) in the striatum between groups. (H) The histogram demonstrates the time for mice retaining on the rod for mice in different groups. (I) Image (left) representing DCX (green) immunoreactivity in the hippocampal tissue sections of mice and histogram (right) indicating the DCX‐positive areas (%) between groups. Scale bars, 100 μm. (J) Image (left) representing Nestin (red) immunoreactivity in the hippocampal tissue sections of mice and histogram (right) indicating the Nestin‐positive areas (%) between groups. Scale bars, 100 μm. (K) Image (left) representing Ki67/NeuN (green and purple) colabeling cells in the hippocampal tissue sections of mice and histogram (right) indicating the Ki67/NeuN‐positive areas (%) between groups. (L and M) The histograms indicate the time (s) for mice to locate a displaced platform (L) and the number of mice crossing the platform (M). Immunohistochemistry analysis: *n* = 4 or 5; behavior analysis: *n* = 8. Scale bars, 100 μm. *, **, and *** represent the significance levels of *p* < 0.05, *p* < 0.01, and *p* < 0.001, respectively, when comparing the PD (Parkinson disease) with Con (control) groups. †, ††, and ††† indicate the significance levels of *p* < 0.05, *p* < 0.01, and *p* < 0.001, respectively, when comparing the PDEx (PD plus exercise) with MPTP‐treated groups. The symbols ψ, ϕ, &, and # indicate that the training effects are significant in the Con, Ex (healthy mice undergoing exercise), PD, and PDEx groups.

Epidemiological data have indicated that MCI is prevalent among patients with PD (Aarsland et al. 2021), but preventive strategies are currently lacking. Emerging evidence from genetic animal models and human postmortem studies of PD shows that exercise can generate beneficial effects on impaired cognition and motor function (Crowley et al. 2019; Hsueh et al. 2018). However, the exact mechanism needs to be further addressed in PD. We, therefore, generated MPTP‐treated mice to explore the mechanism underlying the effects of exercise on PD pathology and cognition (Figure 1A). The MPTP‐treated mice showed increased α‐synuclein, decreased TH‐positive cells, and impaired motor function, suggesting manifested PD pathology (Figure 1B–H). Moreover, the number of DXC^+^, Nestin^+^, and Ki67^+^NeuN^+^ cells was markedly decreased in the hippocampus of MPTP‐treated mice (Figure 1I–K). This indicates that MPTP treatment negatively affected the ability of the hippocampus to produce new neurons and their maturation process. The Morris water maze test indicated longer escape latency and a lower number of platform crossings in MPTP‐treated mice compared to control mice (Figure 1L,M). However, a 10‐week treadmill running program significantly mitigated α‐synucleinopathy, increased TH‐positive cells, and improved motor dysfunction (Figure 1B–H). Moreover, exercise effectively reversed the decreased number of DXC^+^, Nestin^+^, and Ki67^+^NeuN^+^ cells (Figure 1I–K), and led to reduced escape latency and an increased number of platform crossings in MPTP‐treated mice (Figure 1L,M). Together, these results suggest that regular exercise may positively impact PD pathology and, subsequently, enhance neuronal progenitors, immature neurons, and their maturation process, ultimately restoring decreased learning and memory function.

### 2.2 Aerobic Exercise Inhibits Neuronal Apoptosis and Microglial Inflammation in the Hippocampus

**FIGURE 2:**
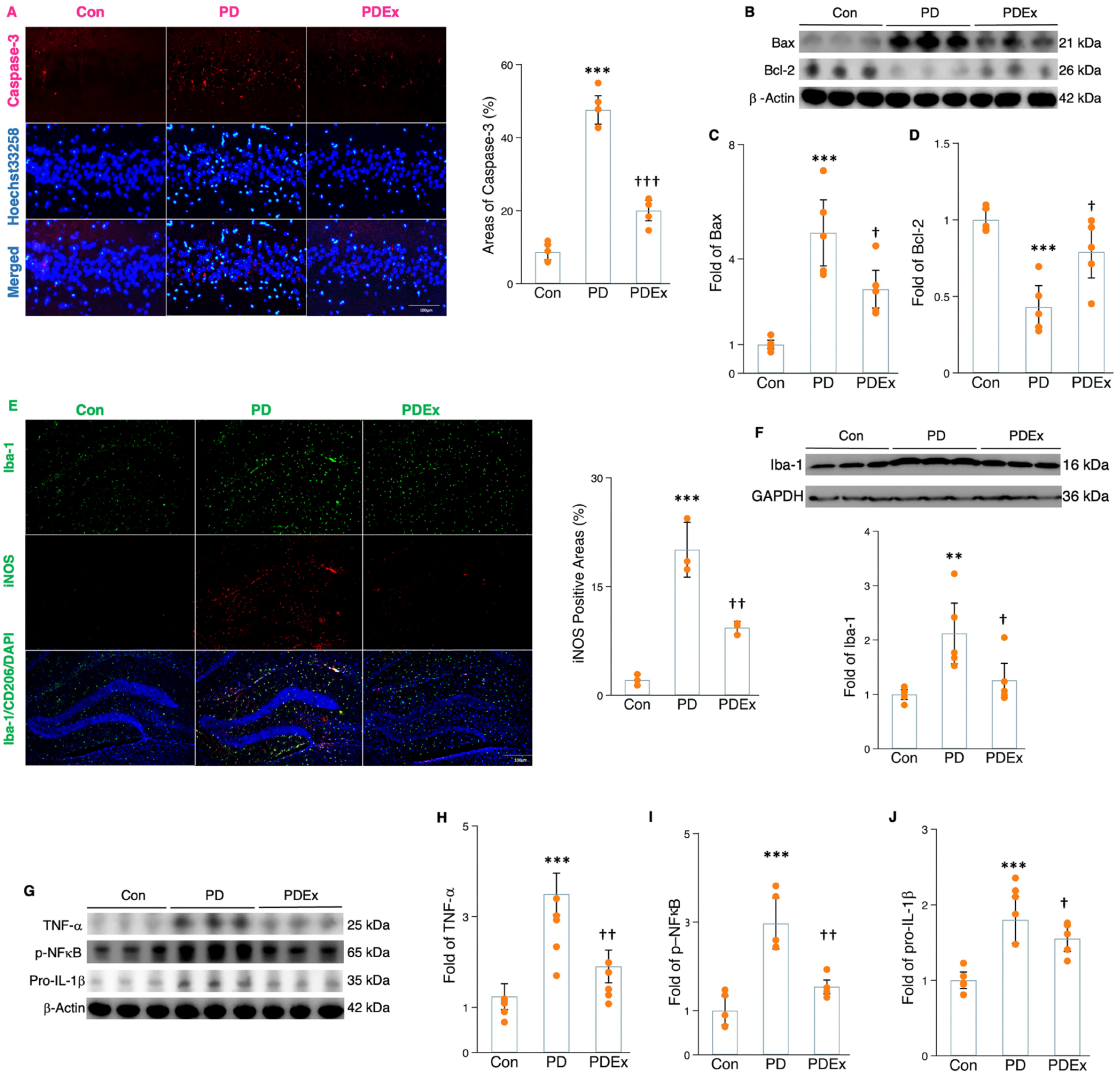
The Impact of exercise on neuronal apoptosis and microglial activation in the hippocampus of MPTP‐treated mice. (A) Image (left) representing fluorescent double staining of caspase‐3 (red) and Hoechst 33258 (light blue) in the hippocampal tissue sections of mice and histogram (right) indicating the caspase‐3‐positive areas (%) between groups. (B, C, and D) Western blot (B) and densitometric analysis for BAX (C) and Bcl‐2 (D) from the hippocampal tissues of mice in different groups. (E) Image (left) representing fluorescent double staining of Iba‐1 (green) and iNOS (red) in the hippocampal tissue sections of mice and histogram (right) indicating the iNOS‐positive areas (%) between groups. (F) Western blot and densitometric analysis for Iba‐1. (G–J) Western blot (G) and densitometric analysis for TNF‐α (H), p‐NFκB (I), and pro‐IL‐1β (J) from the hippocampal tissues of mice in different groups. Western blot analysis: *N* = 4–5 and immunohistochemistry analysis: *N* = 3–4. TNF‐α, Tumor necrosis factor‐alpha. *, **, and *** represent the significance levels of *p* < 0.05, *p* < 0.01, and *p* < 0.001, respectively, when comparing the PD (Parkinson disease) with Con (Control) groups. †, ††, and ††† indicate the significance levels of *p* < 0.05, *p* < 0.01, and *p* < 0.001, respectively, when comparing the PDEx (PD plus exercise) with MPTP‐treated groups.

Fibril α‐synuclein aggregation can lead to neuroinflammation, mitochondrial dysfunction, and subsequent apoptosis (Bloem et al. 2021; Tansey et al. 2022). One major cause of impairment of neurogenesis and memory function is the loss of neurons (Lim et al. 2018). Therefore, we asked whether PD conditions could increase apoptosis of hippocampal neurons and whether exercise could ameliorate these adverse changes. MPTP‐treated mice showed a significant increment in the amount of caspase‐3 fluorescence‐labeling cells, a marker for cellular apoptosis, in the hippocampus (Figure 2A). Moreover, there was an increase in Hoechst 33258‐positive cells in the hippocampus of MPTP‐treated mice, indicating elevated fragmented and densely stained nuclei (Figure 2A). Additionally, MPTP‐treated mice showed elevated levels of proapoptosis marker protein Bax and decreased levels of anti‐apoptosis marker protein Bcl‐2 compared to control mice (Figure 2B–D). However, the excessive apoptosis in the hippocampus of MPTP‐treated mice was decreased after a 10‐week exercise training regimen (Figure 2A–D). Taken together, exercise could generate protective effects on PD‐induced neuronal apoptosis in the hippocampus, which may account for the improvement of AHN and memory in MPTP‐treated mice undergoing exercise intervention.

Neuroinflammation is a hallmark of PD pathology, and fibril α‐synuclein released from dead neurons triggers microglia activity and increases proinflammatory cytokine production, such as Tumor necrosis factor alpha (TNF‐α) (Lee et al. 2019). We proposed that exercise training may have a positive impact on microglial activation and NLRP3 inflammasome priming. In MPTP‐treated mice, there was a greater presence of cells colabeled with Iba‐1 and iNOS (Figure 2E), accompanied by increased Iba‐1 protein expression (Figure 2G). Additionally, the levels of TNF‐α and pro‐IL‐1β were elevated in the hippocampus (Figure 2H,J). However, the increased number of Iba‐1 and iNOS fluorescence labeling cells in the hippocampus and elevated protein expression of Iba‐1, TNF‐α and pro‐IL‐1β could be reversed by 10 weeks of exercise intervention (Figure 2E–J). Furthermore, exercise also decreased p‐NFκB protein expression in the hippocampus when compared to MPTP‐treated mice (Figure 2I). Since TNF‐α and NFκB are key mediators of NLRP3 priming, and pro‐IL‐1β acts as a substrate for NLRP3 inflammasome processing, this implies that PD pathology ‐ activates microglial cells and NLRP3 priming, which could be potentially mitigated by exercise intervention.

### 2.3 Aerobic Exercise Reduced NLRP3 Inflammasome Activation

**FIGURE 3:**
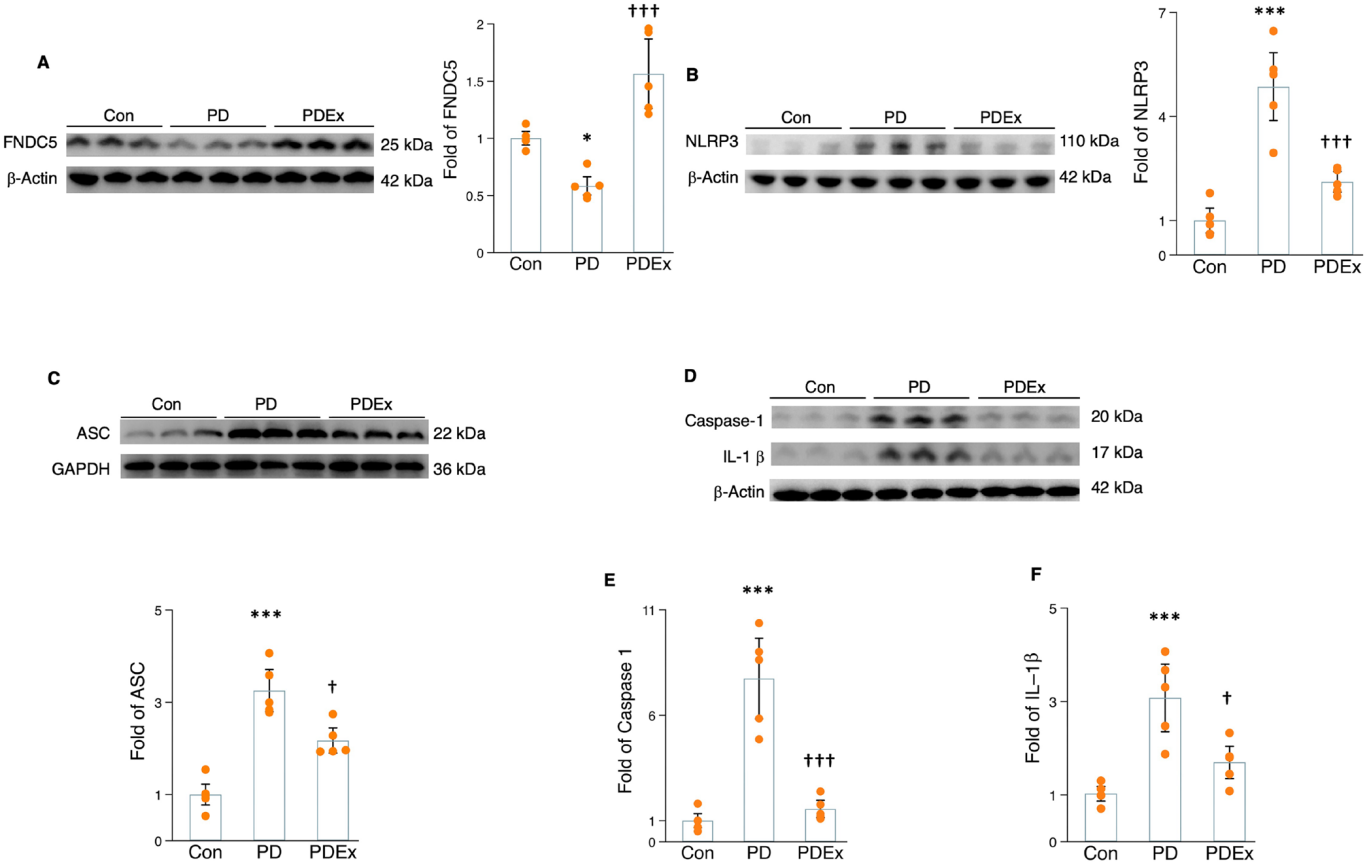
The impact of exercise on protein levels of FNDC5/irisin and NLRP3 components in the hippocampus of MPTP‐treated mice. (A–C) The Western blot and densitometric analysis for FNDC5/irisin (A), NLRP3 (B), and ASC (C). (D) Western blot for caspase‐1 and IL‐1β. (E and F) densitometric analysis for caspase‐1 (E) and IL‐1β (F) from the hippocampal tissues of mice in different groups. Western blot analysis: *N* = 5. *, **, and *** represent the significance levels of *p* < 0.05, *p* < 0.01, and *p* < 0.001, respectively, when comparing the PD (Parkinson disease) with Con (Control) groups. †, ††, and ††† indicate the significance levels of *p* < 0.05, *p* < 0.01, and *p* < 0.001, respectively, when comparing the PDEx (PD plus exercise) with MPTP‐treated groups.

Recent evidence suggests that NLRP3 is a hallmark of PD pathology, contributing to mitochondrial dysfunction and microglial inflammation (Khot et al. 2022; Panicker et al. 2022). NLRP3 has become a major target for addressing PD pathology and associated syndromes, with exercise intervention being one such approach. MPTP‐treated mice showed a remarkable increase in protein levels of NLRP3, ASC, caspase‐1, and Il‐1β in the hippocampus of MPTP‐treated mice (Figure 3B,C,E,F), while the FNDC5/irisin protein expression was markedly downregulated (Figure 3A). However, 10 weeks of treadmill exercise training significantly reduced the protein expressions of NLRP3, ASC, caspase‐1, and Il‐1β (Figure 3B,C,E,F), but increased the FNDC5/irisin protein levels in the hippocampus of MPTP‐treated mice (Figure 3A). Together, these findings suggest that regular exercise not only inhibited NLRP3 inflammasome priming but also decreased its formation in MPTP‐treated mice.

### 2.4 Irisin Decreases Microglial NLRP3 Inflammasome Priming and Formation and IL‐1β Production

**FIGURE 4:**
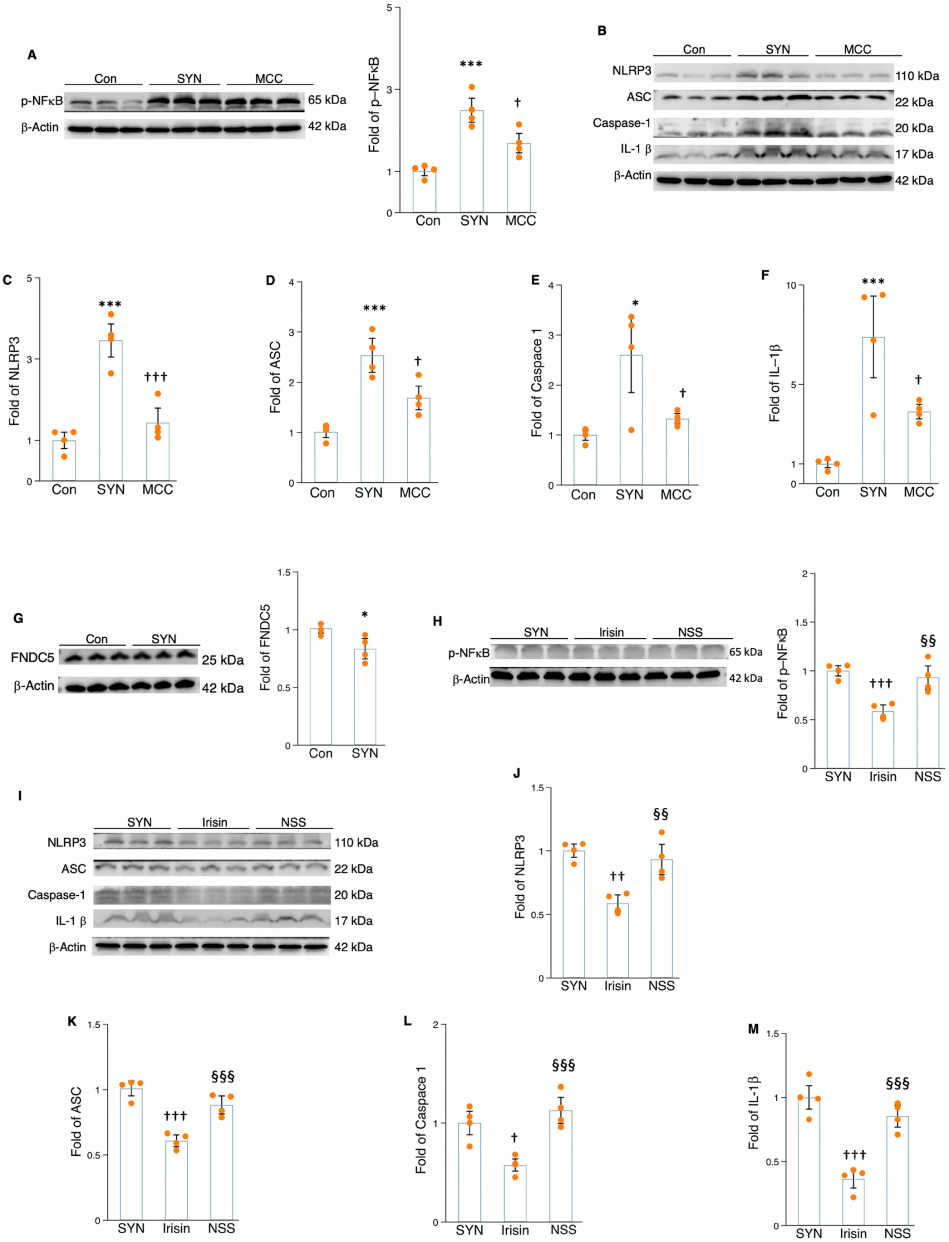
The impact of MCC950 (MC and irisin treatment on the protein levels of NLRP3 components in the α‐synuclein‐cultured microglial cells. (A) The Western blot and densitometric analysis for the impact of MCC950 on p‐NFκB. (B) The Western blot for the impact of MCC950 on NLRP3 components. (C–F) The densitometric analysis for NLRP3 (C), ASC (D), caspase‐1 (E), and IL‐1β (F) in the α‐synuclein‐cultured microglial cells. (G) The Western blot and densitometric analysis for the impact of α‐synuclein on FNDC5/irisin. (H) The Western blot and densitometric analysis for the impact of irisin and NSS treatment on p‐NFκB. (I) The Western blot for NLRP3 inflammasome components. (J–M) The densitometric analysis for the impact of irisin and NSS on NLRP3 (J), ASC (K), caspase‐1 (L), and IL‐1β (M). Western blot analysis: *N* = 4. *, **, and *** represent the significance levels of *p* < 0.05, *p* < 0.01, and *p* < 0.001, respectively, when comparing the SYN (α‐synuclein) with Con (control) groups. †, ††, and ††† indicate the significance levels of *p* < 0.05, *p* < 0.01, and *p* < 0.001, respectively, when comparing MCC (α‐synuclein plus MCC950) and irisin (α‐synuclein plus irisin) with SYN groups. §, §§, and §§§ denote the significance levels of *p* < 0.05, *p* < 0.01, and *p* < 0.001, respectively, when comparing the NSS (α‐synuclein plus irisin and nigericin sodium salt) with irisin groups.

To further validate the findings from the ex vivo experiment, we cultured microglial cells with fibril α‐synuclein for 24 h. Protein expression levels of p‐NFκB, NLRP3, ASC, caspase‐1, and Il‐1β were significantly upregulated by the addition of fibril α‐synuclein into the microglial cell culture (Figure 4A,C–F). The treatment with MCC950, an NLRP3 signaling inhibitor, remarkably reduced the protein levels of NLRP3, ASC, caspase‐1, ‐ IL‐1β, and p‐NFκB expressions (Figure 4A,C–F). This suggests that NLRP3 could be a potential target for addressing microglial inflammation via regulating α‐synuclein‐induced NLRP3 inflammasome activation and IL‐1β secretion. In accordance with the findings observed in brain tissue, protein levels of FNDC5/irisin decreased in microglial cells in the presence of α‐synuclein (Figure 4G). This indicates that irisin may be a protective factor affecting microglial inflammation. Ex vivo addition of recombinant irisin markedly decreased the protein levels of NLRP3 inflammasome priming mediator (NFκB) and its component markers (NLRP3, ASC, and caspase‐1), as well as IL‐1β secretion (Figure 4H,J–M) in α‐synuclein‐cultured microglial cells. However, irisin‐related positive effects on NLRP3 inflammasome components and IL‐1β could be weakened by the administration of the NLRP3 agonist nigericin sodium salt (NSS) (Figure 4J–M). This indicates that irisin signaling can affect both the processes of NLRP3 inflammasome priming and formation, controlling the microglial production of IL‐1β.

### 2.5 Runner Serum Alleviates α‐Synuclein‐Induced Microglial Inflammation via the Irisin/NLRP3 Inflammasome Pathway

**FIGURE 5:**
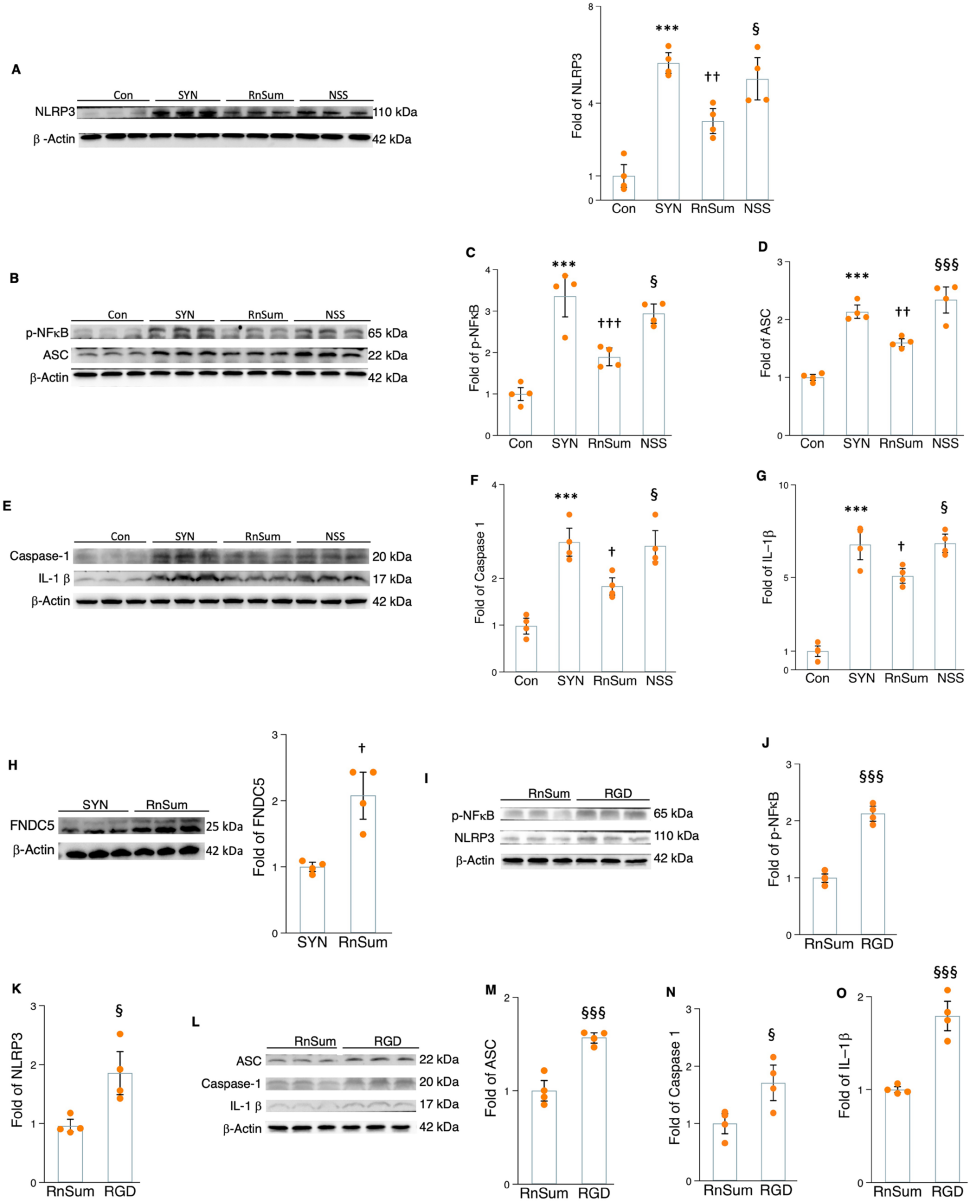
The impact of runner serum, cRGDyk, and NSS on the protein levels of p‐NFκB and NLRP3 components in the α‐synuclein‐cultured microglial cells. (A) The Western blot and densitometric analysis for the impact of runner serum and NSS on NLRP3 in α‐synuclein‐cultured microglia. (B) The Western blot for the impact of runner serum and NSS on p‐NFκB and ASC. (C and D) The densitometric analysis for p‐NFκB (C) and ASC (D). (E) The Western blot for the impact of runner serum and NSS on caspase 1 and IL‐1β. (F and G) The densitometric analysis for caspase‐1 (F) and IL‐1β (G) between groups (*n* = 4). (H) The Western blot and densitometric analysis for the impact of runner serum on FNDC5/irisin in α‐synuclein‐cultured microglia. (I) The Western blot for the impact of runner serum and cRGDyk on p‐NFκB and NLRP3. (J and K) The densitometric analysis for the impact of runner serum and cRGDyk on p‐NFκB (J) and NLRP3 (K). (L) The Western blot for the impact of runner serum and cRGDyk on ASC, caspase‐1, and Il‐1β. (M–O) The densitometric analysis for the impact of runner serum and cRGDyk on ASC (M), caspase‐1 (N), and IL‐1β (O) between groups. Western blot analysis: *N* = 4. *, **, and *** represent the significance levels of *p* < 0.05, *p* < 0.01, and *p* < 0.001, respectively, when comparing the SYN (α‐synuclein) with Con (control) groups. †, ††, and ††† indicate the significance levels of *p* < 0.05, *p* < 0.01, and *p* < 0.001, respectively, when comparing the RnSum (α‐synuclein plus Runner serum) with SYN groups. §, §§, and §§§ denote the significance levels of *p* < 0.05, *p* < 0.01, and *p* < 0.001, respectively, when comparing the NSS (α‐synuclein plus RnSum and nigericin sodium salt) and RGD (α‐synuclein plus RnSum and cyclo RGDyk) with RnSum groups.

We further examined the potential mechanism by which exercise affects microglial NLRP3 inflammasome activation. The beneficial effects of exercise on the biological and physiological functions of organs are believed to be linked to the production of hundreds of myokines (Horowitz et al. 2020; Zhao 2024a; Zhao 2024b). These myokines are produced during exercise, circulate in the bloodstream, and enter various organs, where they exert their effects. Recent studies have indicated that blood containing major exercise molecules may partially replicate the effects of exercise on brain plasticity and function (Horowitz et al. 2020; Zhao 2024a; Zhao 2024b). First, we collected and separated the serum from rats undergoing a 4‐week treadmill running program and then added runner serum into α‐synuclein‐cultured microglia cells (incubation for 24 h). Runner serum significantly reduced the protein levels of p‐NFκB, NLRP3, ASC, caspase‐1, and IL‐1β (Figure 5A,C,D,F,G) in microglial cells. However, treatment of NSS decreased the anti‐inflammatory effects on NLRP3 inflammasome components and IL‐1β levels (Figure 5A,C,D,F,G). This suggested that exercise serum could reduce the processes of microglial NLRP3 inflammasome formation, as well as cytokine production. Interestingly, runner serum increased the FNDC5/irisin levels in α‐synuclein‐cultured microglial cells (Figure 4H). In combination with our in vivo data, it is indicated that FNDC5/irisin signaling may be a potential mediator for the exercise‐related anti‐inflammation effects. We added cyclo RGDyk (cRGDyk) into the α‐synuclein‐cultured microglial cells in the presence of runner serum. The anti‐inflammatory effects of runner serum, including reduced protein levels of p‐NFκB, NLRP3, ASC, caspase‐1, and IL‐1β, could be diminished by treatment with cRGDyk (Figure 5J,K,M–O). Together, these findings indicate that exercise serum has positive effects on microglial NLRP3 inflammasome activation and cytokine production by upregulating FNDC5/irisin signaling.

### 2.6 Blocking Irisin Signaling Diminished the Neuroprotective Effects of Exercise on MPTP‐Treated Mice

**FIGURE 6:**
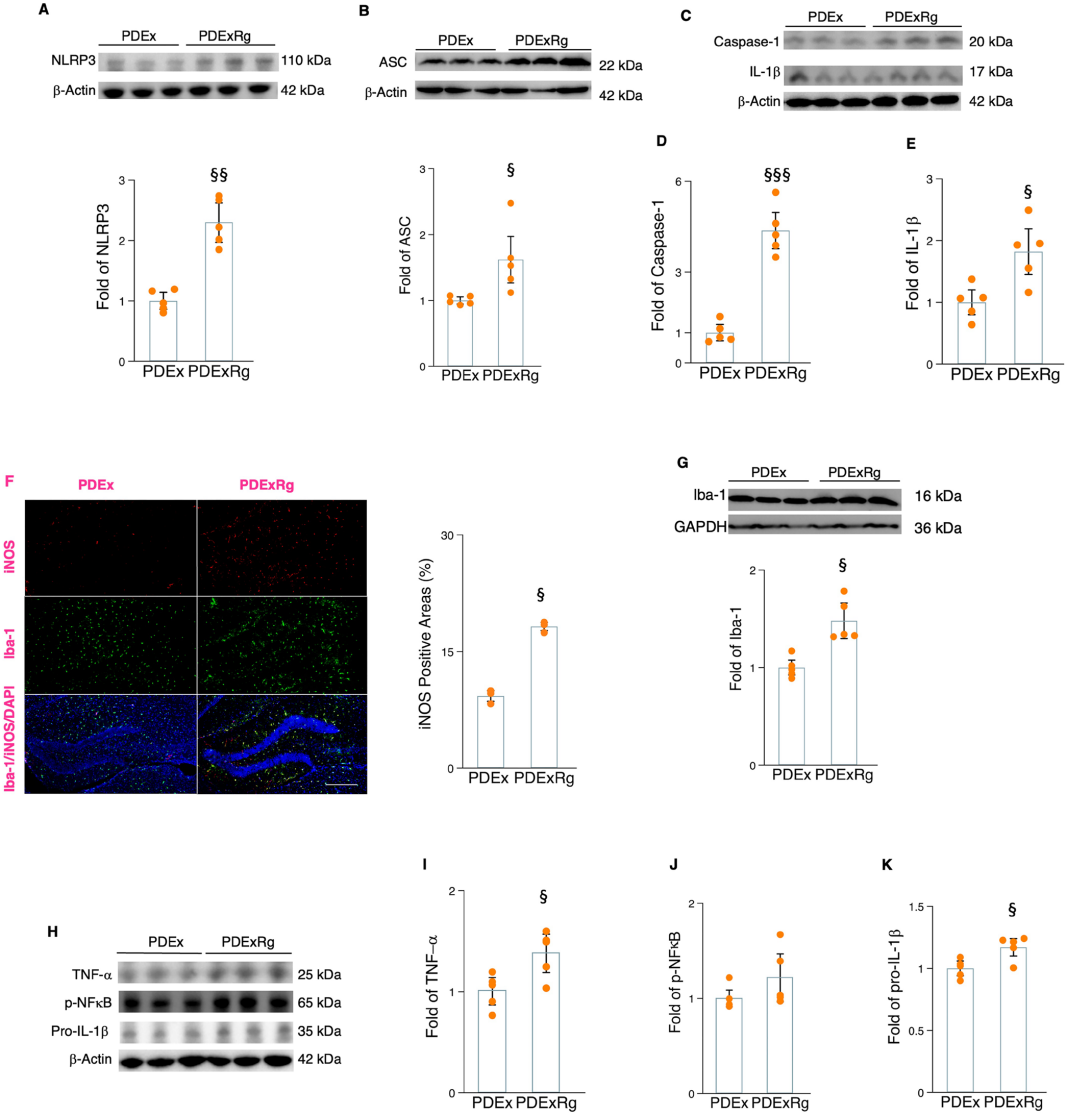
Blocking irisin signaling diminished the neuroprotective effects of exercise on microglial activation and NLRP3 inflammasome formation in MPTP‐treated mice. (A and B) The Western blot and densitometric analysis for the impact of cRGDyk administration on exercise‐induced changes in NLRP3 (A) and ASC (B) in MPTP‐treated mice between groups. (C) Western blot for caspase‐1 and IL‐1β. (D and E) densitometric analysis for caspase‐1 (D) and IL‐1β (E) from the hippocampal tissues of mice in different groups. (F) Image (left) representing fluorescent double staining of Iba‐1 (green) and iNOS (red) in the hippocampal tissue sections of mice and histogram (right) indicating the iNOS‐positive areas (%) between groups. (G) Western blot and densitometric analysis for Iba‐1. (H–K) Western blot (H) and densitometric analysis for TNF‐α (I), p‐NFκB (J), and pro‐IL‐1β (K) from the hippocampal tissues of mice in different groups. Western blot analysis: *N* = 5 and immunohistochemistry analysis: *N* = 4. §, §§, and §§§ denote the significance levels of *p* < 0.05, *p* < 0.01, and *p* < 0.001, respectively, when comparing the PDExRg (Parkinson disease plus exercise and cyclo RGDyk) with PDEx (PD plus exercise) groups.

We next examined the role of irisin signaling in mediating the positive effects of exercise on microglial inflammation, NLRP3 inflammasome activation, and cytokine secretion in MPTP‐treated mice. While exercise enhanced FNDC5/irisin protein levels and reduced expressions of p‐NFκB, NLRP3 inflammasome components, and IL‐1β, a 10‐week treatment of cRGDyk reversed these changes in the hippocampus of MPTP‐treated mice (Figure 6A,B,D,E). The number of Iba‐1‐iNOS‐labeled microglial cells and Iba‐1 protein levels were increased in running MPTP‐treated mice administered cRGDyk (Figure 6F,G). Furthermore, protein levels of TNF‐α and pro‐IL‐1β were also elevated in the hippocampus (Figure 6I,K). These findings suggest that irisin signaling may contribute to the beneficial effects of exercise on neuroinflammation by reducing microglial activation and NLRP3 inflammasome formation.

**FIGURE 7:**
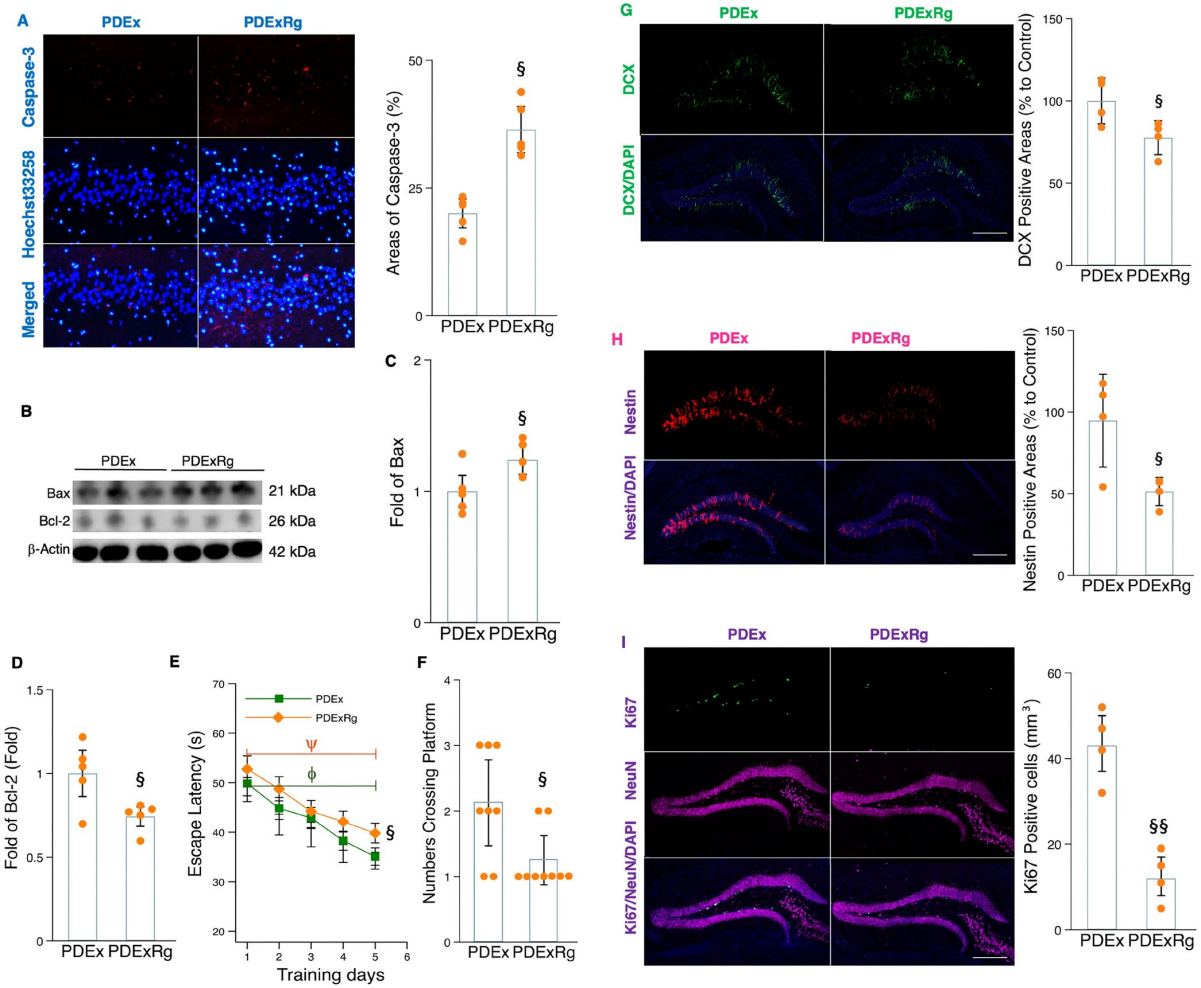
Blocking irisin signaling decreased the effects of exercise on cell apoptosis, AHN, and memory in MPTP‐treated mice. (A) Image (left) representing fluorescent double staining of caspase‐3 (red) and Hoechst 33258 (light blue) in the hippocampal tissue sections of mice and histogram (right) indicating the caspase‐3‐positive areas (%) between groups. (B, C, and D) Western blot (B) and densitometric analysis for BAX (C) and Bcl‐2 (D) from the hippocampal tissues of mice in different groups. (E and F) The histograms indicate the time (s) for mice to locate a displaced platform (E) and the number of crossings of the platform (F). (G) Image (left) representing DCX (green) immunoreactivity in the hippocampal tissue sections of mice and histogram (right) indicating the DCX‐positive areas (%) between groups. Scale bars, 100 μm. (H) Image (left) representing Nestin (red) immunoreactivity in the hippocampal tissue sections of mice and histogram (right) indicating the Nestin‐positive areas (%) between groups. Scale bars, 100 μm. (I) Image (left) representing Ki67/NeuN (green and purple) colabeling cells in the hippocampal tissue sections of mice and histogram (right) indicating the Ki67/NeuN‐positive areas (%) between groups. Western blot analysis: *N* = 5; immunohistochemistry analysis: *N* = 4; behavior analysis: *N* = 8. Scale bars, 100 μm. §, §§, and §§§ denote the significance levels of *p* < 0.05, *p* < 0.01, and *p* < 0.001, respectively, when comparing PDExRg (PD plus exercise and cyclo RGDyk) with PDEx (PD plus exercise) groups. The symbols ψ and ϕ indicate that the training effects are significant in the PDEx and PDExRg groups.

We further determined the role of irisin signaling in mediating exercise‐related improvements in neuronal apoptosis and AHN in the hippocampus of MPTP‐treated mice. In comparison with the exercise training group, the administration of cRGDyk led to an increase in the presence of caspase‐3 and Hoechst 33258 fluorescence‐labeled cells in the hippocampus of MPTP‐treated mice (Figure 7A). Accordingly, the protein levels of Bax increased, while the expression of Bcl‐2 decreased following cRGDyk administration in MPTP‐treated mice undergoing treadmill running (Figure 6C,D). These findings indicate that cRGDyk may inhibit the beneficial effects of exercise on PD neuropathology by increasing neuronal apoptosis and microglial inflammation in the hippocampus of MPTP‐treated mice. Moreover, compared to the exercise intervention group, cRGDyk treatment decreased the number of DCX^+^, Nestin^+^, and Ki67^+^NeuN^+^ cells within the hippocampus of MPTP‐treated mice (Figure 7G–I). Similarly, escape latency was significantly increased while the number of crossings of the platform was markedly reduced in MPTP‐treated mice that received cRGDyk during exercise training (Figure 7E,F). These findings suggest that irisin signaling emerges as a key mediator of exercise‐related improvements in microglial activation, cell apoptosis, and inflammation, as well as the enhancement of AHN and memory function in MPTP‐treated mice.

### 2.7 Irisin Signaling Could be a Pharmacological Target for Enhancing AHN and Memory Function

**FIGURE 8:**
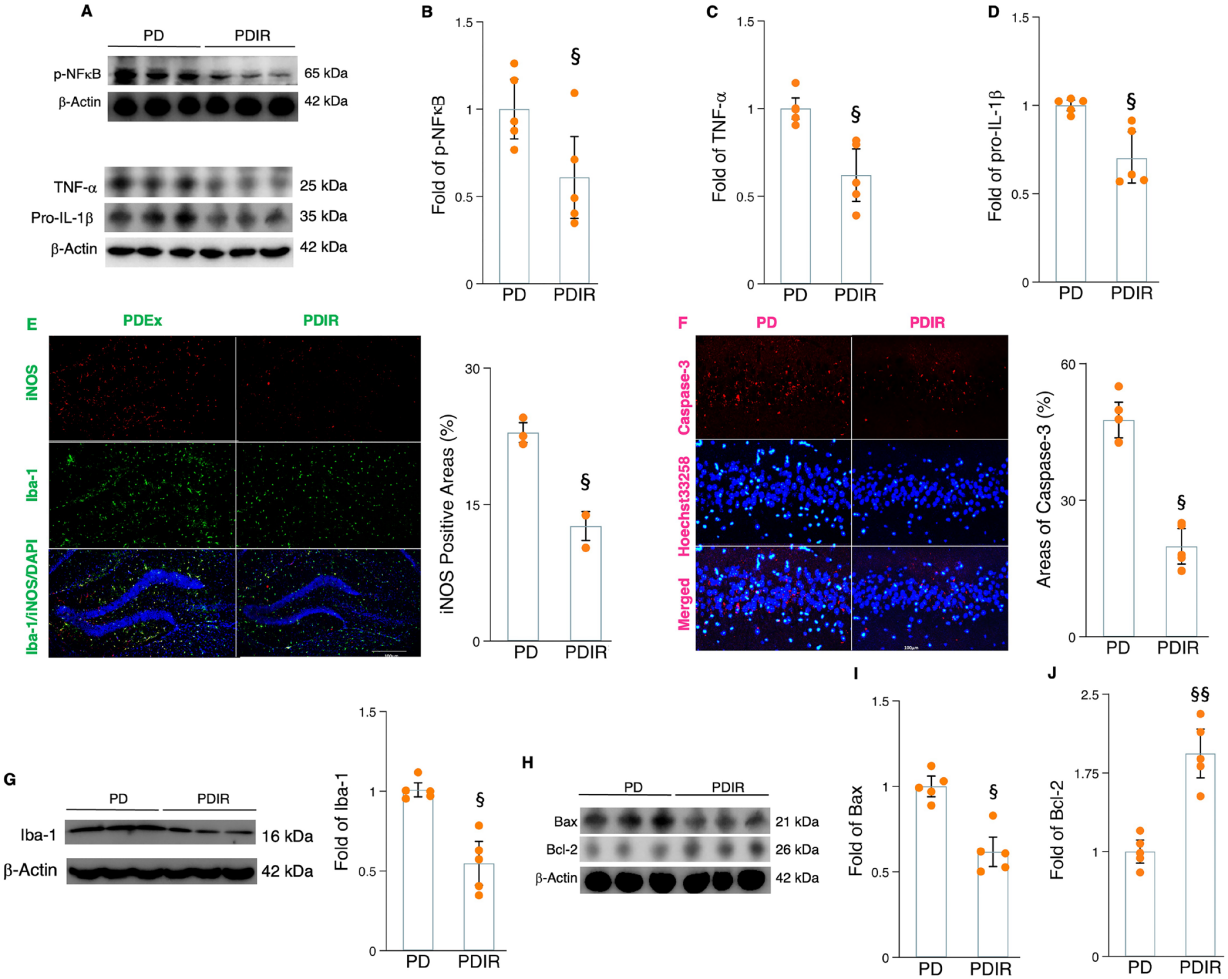
The effects of recombinant irisin on microglial activation, inflammatory cytokines, and neuronal apoptosis in MPTP‐treated mice. (A–D) Western blot (A) and densitometric analysis for TNF‐α (B), p‐NFκB (C), and pro‐IL‐1β (D) from the hippocampal tissues of mice in different groups. (E) Image (upper) representing fluorescent double staining of Iba‐1 (green) and iNOS (red) in the hippocampal tissue sections of mice and histogram (lower) indicating the iNOS‐positive areas (%) between groups. (F) Image (upper) representing fluorescent double staining of caspase‐3 (red) and Hoechst 33258 (light blue) in the hippocampal tissue sections of mice and histogram (lower) indicating the caspase‐3‐positive areas (%) between groups. (G) Western blot and densitometric analysis for Iba‐1. (H–J) Western blot (H) and densitometric analysis for BAX (I) and Bcl‐2 (J) from the hippocampal tissues of mice in different groups. Western blot analysis: *N* = 5 and immunohistochemistry analysis: *N* = 4. §, §§, and §§§ denote the significance levels of *p* < 0.05, *p* < 0.01, and *p* < 0.001, respectively, when comparing the PDIR (PD plus irisin) with MPTP‐treated groups.

**FIGURE 9:**
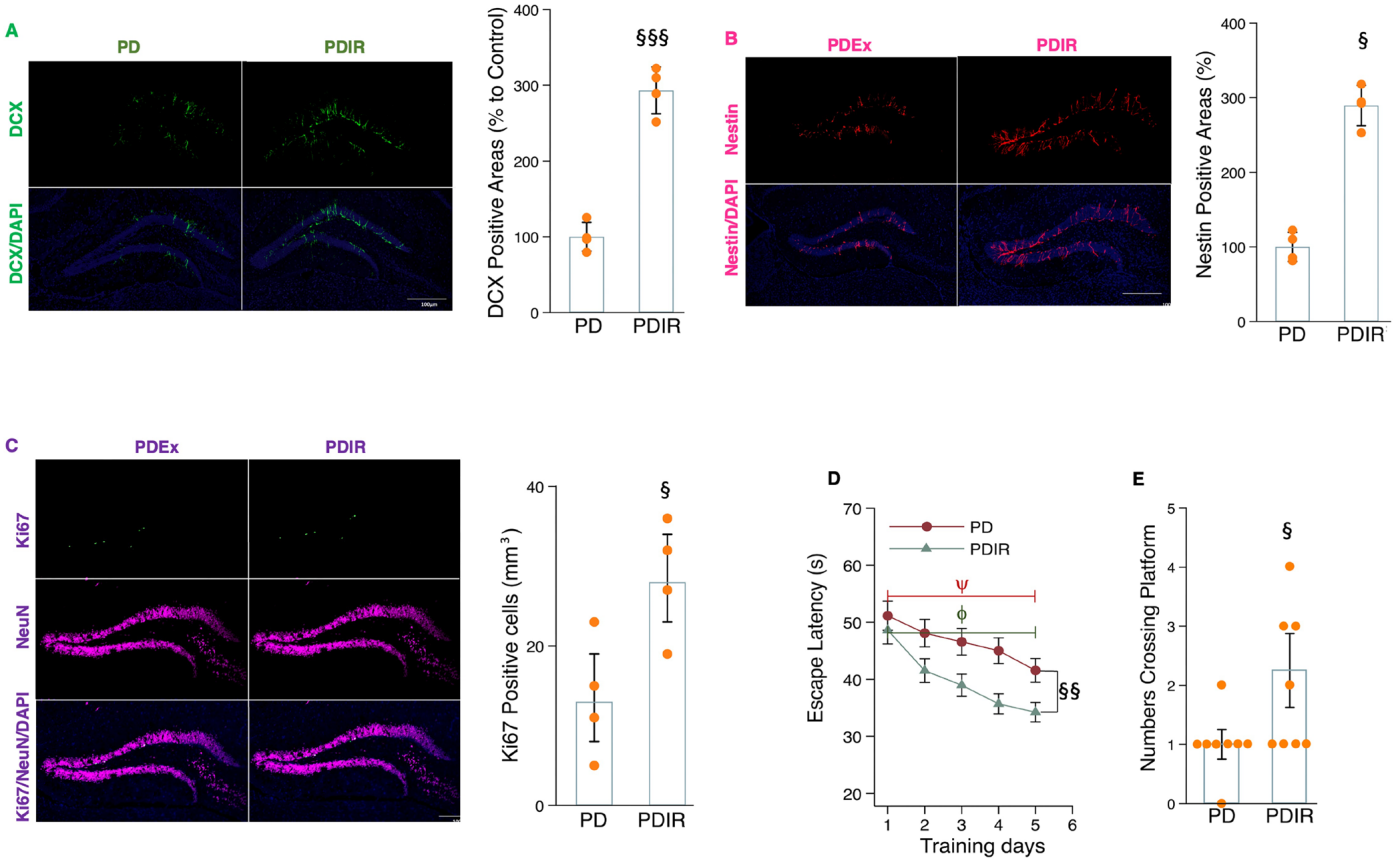
The effects of recombinant irisin treatment on adult hippocampal neurogenesis and memory performance in MPTP‐treated mice. (A) Image (left) representing DCX (green) immunoreactivity in the hippocampal tissue sections of mice and histogram (right) indicating the DCX‐positive areas (%) between groups. Scale bars, 100 μm. (B) Image (left) representing Nestin (red) immunoreactivity in the hippocampal tissue sections of mice and histogram (right) indicating the Nestin‐positive areas (%) between groups. Scale bars, 100 μm. (C) Image (left) representing Ki67/NeuN (green and purple) colabeling cells in the hippocampal tissue sections of mice and histogram (right) indicating the Ki67/NeuN‐positive areas (%) between groups. (D and E) The histograms indicate the time (s) for mice to locate a displaced platform (D) and the number of crossings of the platform (E). Immunohistochemistry analysis: *N* = 4; behavior analysis: *N* = 8. Scale bars, 100 μm. §, §§, and §§§ denote the significance levels of *p* < 0.05, *p* < 0.01, and *p* < 0.001, respectively, when comparing the PDIR (PD plus irisin) with MPTP‐treated groups. The symbols ψ and ϕ indicate that the training effects are significant in the PD and PDIR groups.

Given that irisin signaling is a crucial modulator of exercise‐related protective effects on neuroinflammation, AHN, and cognition, we further determined whether this molecule could be a pharmacological target for reducing PD pathology and enhancing AHN and cognition in MPTP‐treated mice. Ten weeks of recombinant irisin administration led to decreased levels of TNF‐α, p‐NFκB, and pro‐IL‐1β proteins (Figure 8B–D), while the number of Iba‐1‐iNOS labeling microglial cells and Iba‐1 protein expression significantly reduced in the hippocampus of MPTP‐treated mice (Figure 8E,G). Compared to the MPTP‐treated group, a 10‐week treatment with recombinant irisin significantly decreased the number of caspase‐3 and Hoechst 33258‐positive cells in the hippocampus of MPTP‐treated mice (Figure 8F). Additionally, irisin administration led to a decrease in Bax protein levels and an increase in Bcl‐2 expression in MPTP‐treated mice (Figure 8I,J). Those findings collectively indicate that irisin shows promise in mitigating neuroinflammation and apoptosis in MPTP‐treated mice. Additionally, it was observed that irisin treatment increased the populations of Nestin^+^, DCX^+^, and Ki67^+^NeuN^+^ cells in the hippocampus of MPTP‐treated mice (Figure 9A–C). Consequently, escape latency decreased while the number of platform crossings increased in MPTP‐treated mice administering irisin (Figure 9D,E). These results suggest that recombinant irisin may replicate some of the neuroprotective benefits of exercise by inhibiting microglial inflammation and apoptosis, thereby enhancing AHN and cognition in PD.

## 3 Discussion

This study has shown that aerobic exercise can be an effective strategy for enhancing hippocampal neurogenesis AHN and memory function in MPTP‐treated mice. The neuroprotective effects induced by exercise are partially mediated through the inhibition of microglial NLRP3 inflammasome activation and neuronal apoptosis via the irisin signaling pathway. Administering recombinant irisin shows favorable impacts on hippocampal inflammation and improves AHN and memory function, offering a potential approach for addressing PD pathology and related symptoms.

Currently, PD is regarded as an incurable disease, and there are no effective strategies for addressing cognitive deficits. Recently, clinical data suggest that regular exercise generates multiple benefits on cognitive function in PD patients (Murray et al. 2014). Exercise can promote hippocampal neurogenesis and enhance memory consolidation and forgetting (Lu et al. 2023), providing a therapeutic approach to improving AHN and cognitive function in health and diseases. However, the impact and underlying mechanisms of exercise on enhancing AHN and subsequent cognitive improvement are not yet fully addressed in PD. Physical exercise can bring about various positive effects on the brain, such as enhancing the secretion of growth factors like brain‐derived neurotrophic factor (BDNF) that aid in neuronal survival, differentiation, and integration, as well as boosting brain blood flow to supply vital nutrients and oxygen for neurogenesis (Lu et al. 2023; Zhao 2024b). Our findings indicated that exercise not only reduces PD pathology, such as the accumulation of α‐syn and the loss of DAergic neurons but also reduces microglial activation, proinflammatory cytokine production, and neuronal apoptosis. Recent evidence supports that exercise has anti‐inflammatory and anti‐apoptotic effects in neurodegenerative conditions (Jang et al. 2018; Lin et al. 2020; Wang et al. 2023b), potentially protecting against neurodegeneration and benefiting AHN and cognition. Although there is evidence suggesting that exercise may reduce NLRP3 inflammasome activation in heart disease and AD (Ahmadi et al. 2022; Rosa et al. 2021), the specific effects of exercise on the NLRP3 inflammasome in PD remain unclear. Our findings indicate that exercise or runner serum can decrease levels of TNF‐α, NF‐κB, NLRP3, and pro‐IL‐1β—key markers of NLRP3 inflammasome priming—in the hippocampus of MPTP‐treated mice or α‐synuclein‐cultured microglial cells. This indicates that exercise may affect the priming processes of the NLRP3 inflammasome. Additionally, exercise also reduces the expression of ASC, caspase‐1, and IL‐1β, suggesting it inhibits the formation processes of the NLRP3 inflammasome.

Recent studies have shown that transferring blood from exercised mice to older or diseased mice can help maintain brain plasticity and function (Choi et al. 2018; de Miguel et al. 2021; Horowitz et al. 2020). These beneficial effects are thought to arise from the presence of various molecules, such as irisin and BDNF, in the plasma of exercised mice. These molecules are produced during exercise, enter the bloodstream, and influence organ function (Pedersen 2019; Wrann et al. 2013). However, in ex vivo cell culture experiments, plasma—unlike serum—lacks the growth factors and nutrients necessary to support cell growth. In contrast, serum contains a wide range of exercise‐induced molecules and effectively promotes cell growth. For this reason, we selected serum as a supplement for our cell culture studies. The use of serum in cell culture is a common practice in other research areas, including studies on cell viability, migration, and proliferation (de Cabo et al. 2015; Vianna et al. 2015; Wu et al. 2017).

The anti‐inflammatory effects of exercise may be regulated by irisin. Accumulation of α‐synuclein negatively impacts irisin levels; however, both regular exercise and serum from exercising individuals can restore these levels and reduce NLRP3 inflammasome activation in the hippocampus of MPTP‐treated mice and α‐synuclein‐cultured microglia. These anti‐inflammatory effects are linked to irisin signaling, as treatment with cRGDyk, either in vivo or ex vivo, diminishes these beneficial effects. This suggests that irisin signaling plays a critical role in mediating the effects of exercise in PD. Furthermore, ex vivo treatment of microglia with irisin alleviated α‐synuclein‐induced activation of NLRP3 inflammasome, while in vivo administration of irisin in MPTP‐treated mice reduced microglial inflammation and NLRP3 inflammasome activation, leading to improved AHN and memory performance. Thus, the irisin/NLRP3 signaling pathway may be a key mediator of the protective effects of exercise on hippocampal inflammation, cell apoptosis, adult neurogenesis, and cognitive function in PD. Given the absence of effective medications for addressing cognitive deficits in PD, our findings that 10 weeks of irisin administration effectively reduced hippocampal inflammation and improved AHN and memory function may provide viable strategies to address these cognitive challenges.

In summary, our study presents both in vivo and ex vivo evidence demonstrating the role of exercise in enhancing AHN and cognitive functions by reducing α‐synucleinopathy, neuroinflammation, and apoptosis in the hippocampus of PD. Our findings suggest that the irisin/NLRP3 inflammasome signaling pathway may play an essential role in these beneficial effects. However, several limitations should be noted. For example, our study focused solely on the relationship between irisin and the NLRP3 inflammasome in mediating the neuroprotective effects of exercise in MPTP‐treated mice. Exploring additional PD models, such as transgenic mouse models, could strengthen the evidence. Additionally, our research included only male mice to assess the impact of exercise on PD pathology. This highlights the need to include both male and female mice in future studies, which could yield valuable insights into sex differences in response to exercise interventions. Moreover, in our ex vivo cell culture experiments, we used serum derived from running rats for incubation. In future research, we plan to incorporate both rat and mouse serum for cell culture experiments to broaden the scope of our findings. Despite these limitations, our results suggest a promising nonpharmacological approach for treating PD pathology and improving cognitive function.

## 4 Methods

### 4.1 Animals

The Animal Care and Use Committee of Yangzhou University approved the protocols for the use of male wild‐type C57BL/6J mice (No. 202303133), which were 8 weeks old and purchased from the experimental animal center of Yangzhou University, Yangzhou, China. Four animals were accommodated per cage in the university's animal facility, where they were maintained at a temperature of 23°C ± 2°C and subjected to a 12‐h light–dark cycle for at least 1 week prior to their use for the designated experiment. Forty C57BJ/6 J mice were randomly divided into five groups: control (Con), MPTP‐treated (PD), MPTP treated with exercise (PDEx), MPTP treated with exercise and cRGDyk (PDExRg), and MPTP treated with irisin (PDIR).

### 4.2 Exercise Protocols and Drug Administration

To induce PD pathology in mice, we used a subacute MPTP (Sigma‐Aldrich, St. Louis, MO, USA) regimen. Mice received 30 mg/kg free‐base MPTP intraperitoneally (i.p.) daily for 5 consecutive days, following previous protocols (Jackson‐Lewis and Przedborski 2007) (Figure 1A). We used sterile saline as a vehicle and injected control mice with saline using the same regimen. We defined these mice as sham.

During exercise training, mice in the PDExRg group were administered a tail intravenous injection of cRGDyk (agent for blocking αVβ5) (GLPBIO Biological Company, USA) at a dosage of 2.5 mg/kg twice a week for 10 weeks. Those mice in the PDIR group received twice‐weekly tail injections of recombinant irisin (PeproTech Bio, USA) at a dosage of 100 μg/kg for 10 weeks. In contrast, the other groups received the same amount of 10% dimethylsulfoxide solution.

The mice underwent a 10‐week aerobic treadmill protocol, running 5 days a week. During the 1st week, they acclimatized to the treadmill by gradually increasing their speed from 8 to 12 m/min and their running time from 10 to 60 min. Subsequently, for the following weeks, they consistently ran for 60 min at a speed of 12 m/min during each workout session (Figure 1A).

### 4.3 Locomotor Evaluation

To assess the locomotor function of mice exposed to MPTP, we performed the rotarod test (Ugo Basile, Gemonio, Italy) using a 3‐cm cylinder rod with an acceleration program. The mice received 3 days of training before MPTP administration. On the test day, the mice were put on the rotarod device, which started at 1 rpm and followed a 12 rpm/2 s protocol until reaching 50 rpm. We measured the time and speed of the mice falling off the rotating bar. The maximum time on the rod was 300 s. We calculated the results as the mean of three trials, with a 40‐min interval between each trial.

### 4.4 Morris Water Maze Test

The Morris water maze was performed after the finish of the experiment as described previously (Hosseini‐Sharifabad et al. 2011). After 1 day of habituation, mice began a 6‐day training course in a submerged platform version of the water maze. The learning test was conducted on the first 5 days, and the memory test was conducted on the 6th day. For the learning test, the rodents were placed in the pool at different starting points and found the hidden platform. The time taken to find the platform (escape latency) was recorded. The learning test ended when the mice reached the platform or after 60 s had passed. On the 6th day, the platform was removed, and the test was conducted for 60 s to observe how many times the mice crossed the original platform location while swimming.

### 4.5 Microglial Cell Culture and Treatment

BV2 microglial cells (Procell Life, CL‐0493A) incubated in DMEM (Servicebio, G4520, China) containing 10% fetal bovine serum (FBS; Vazyme, F101–01, China) and 1% penicillin–streptomycin maintained at 37°C in a humidified incubator with 5% CO_2_. Subsequently, the medium was replaced, and preformed fibril (PFF) α‐synuclein (1 μM) was added to the cell culture and incubated for 24 h. After this, the cells were collected for analysis. To further investigate the effects of exercise on microglial activity and NLRP3 inflammasome components, serum was separated from rats that had undergone 4 weeks of treadmill running exercise. Subsequently, the “runner serum” was added to the BV2 cell culture system for 24 h, followed by interventions with irisin, cRDGyk, MCC950 (MedChemExpress, New Jersey, USA), or NSS (MedChemExpress, New Jersey, USA).

### 4.6 Serum Preparation and Treatment

Healthy Wistar male rats (aged 2 months) underwent either treadmill training or maintained their routine activity for 4 weeks. Both the running and control groups (10 rats per group) were euthanized between 07:00 a.m. and 09:30 a.m. with 18 μL of a 2.5% solution of avertin per gram of body weight (Sigma‐Aldrich, T48402) for blood collection. Blood was collected from the right heart ventricle and allowed to clot at room temperature for approximately 30 min. The clotted blood was then centrifuged at 5000 g for 20 min. The serum layer was carefully extracted using a pipette, filtered through a 0.22 μm sterile syringe filter, and then stored at −80°C. In the exercise serum treatment experiment, α‐synuclein‐cultured microglial cells were treated with a mixture of 10% exercise serum, 1% penicillin–streptomycin, and 89% DMEM, while serum from sedentary rats was used as a control supplement added to these culture systems.

### 4.7 Immunohistochemistry and Immunofluorescence Staining

Mice were anesthetized as described previously and then perfused with 20 mL of 1 M phosphate‐buffered saline (PBS). The left hemispheres were preserved in 4% paraformaldehyde at 4°C for 48 h, followed by immersion in a 30% sucrose solution for an additional 48 h to prepare for brain sectioning. Serial coronal sections (thickness, 20 μm) were obtained using a freezing microtome (Leica, CM1950) and kept at −80°C until further analysis. Immunohistochemistry and immunofluorescent labeling were performed as described previously (Choi et al. 2008). The brain hemispheres were serially sectioned at 4 μm in the SN, ST, or hippocampus regions according to the 3D brain stereotaxic atlas. We performed endogenous peroxidase blocking, then blocked the sections with 10% goat serum for 1 h. For immunohistochemistry, the sections were treated with TH antibody (1:1000, GB11181, Servicebio, China) and α‐syn (1:500, sc‐13,757, Santa, USA) for 16 h at 4°C, then with a secondary antibody for 40 min at room temperature. For immunofluorescence staining, we followed the same procedure with the subsequent primary antibodies: NeuN (1:1000, GB11138, Servicebio, China), DCX (1:50, sc‐271,390, Santa Cruz Biotechnology, USA), Nestin (1:200, Ab221660, Abcam, USA), Ki67 (1:200, TW0001, Abmart, China), caspase‐3 (1:50, sc‐56,053, Santa, USA), Iba‐1 (1:100, AF7143, Beyotime, China), and iNOS (1:50, sc‐7271, Santa, USA), followed by fluorescence‐labeled secondary antibodies. Finally, DAPI/Hoechst 33258 was added dropwise to aid in visualizing the nuclei. The sections were observed and acquired using an ultrahigh‐resolution confocal laser microscope (TCS SP8 STED, Leica, Germany). The mean intensity of fluorescence was measured using Image J software (version 6.0, Media Cybernetics, USA).

### 4.8 Western Blot Analysis

Western blot analysis was conducted by lysing the mouse SN or hippocampus in lysis buffer to extract the total protein, which was then subjected to SDS‐PAGE. The transferred membranes were subsequently blocked in 5% skimmed milk for 2 h at room temperature. Following this, the membranes were incubated for 18 h at 4°C with a range of antibodies, including FNDC5 (1:1000, A18107, Abclonal, Wuhan, China), NFκB (1:500, sc‐8008, Santa Cruz, CA, USA), p‐NFκB (1:1000, sc‐136,548, Santa Cruz, USA), NLRP3 (1:1000, BF8029, Affinity Biosciences, China), ASC (1:500, NBP2‐12446, Novus, USA), proIL1β (1:500, AF5103, Affinity Biosciences, China), IL‐1β (1:200, WL00891, Wanleibio, China), Iba‐1 (1:1000, AF7143, Beyotime, China), TNF‐α (1:500, sc‐12,744, Santa, USA), caspase‐1 (1:1000, 22,915‐1‐AP, Proteintech, China), Bax (1:1000, WL01637, Wanlei, China), Bcl‐2 (1:1000, WL01556, Wanlei, China), caspase‐3 (1:500, sc‐56,053, Santa, USA), and β‐actin (1:50000, 66,009‐1‐lg, Proteintech, China). After this incubation, the membranes were further incubated with specific secondary antibodies, goat anti‐rabbit IgG (1:10000, BF03008, Biogragon, China), or goat anti‐mouse IgG (1:10000, BF03001, Biogragon, China), for 1–1.5 h. The density of protein bands was evaluated with Image‐Pro Plus software (version 6.0, Media Cybernetics, USA) and normalized to the equivalent density of the housekeeping protein β‐actin.

### 4.9 Statistics

All the analyses were conducted by STATA software (Version 15, StataCorp LP, Texas, USA). Data were reported as mean and standard deviation (SD). Student t‐test was used to analyze the difference between the two groups. A one‐way analysis of variance (ANOVA) with the Bonferroni post hoc test was used for multiple comparisons among groups. For evaluating both training and treatment effects, a two‐way ANOVA was applied for analysis. A level of *p* < 0.05 was accepted as significant.

## Author Contributions

R.Z. and X.T.: Conceptualization and design of the study; X.T., H.X., B.W., Y.W., and J.L.: conduction of the experiment, acquisition and analysis of data, and preparation of figures; R.Z.: writing – original draft and writing – review and editing; and X.T., H.X., B.W., Y.W., and J.L.: writing – review and editing.

## Ethics Statement

Our studies did not include human participants or human tissue. All animals used in the study are approved by the Animal Care and Use Committee of Yangzhou University.

## Consent

The authors consent to the publication of this study.

## Conflicts of Interest

The authors declare no conflicts of interest.

## Data Availability

The data that support the findings of this study are available on request from the corresponding author. The data are not publicly available due to privacy or ethical restrictions.
